# Drug-Drug Interactions Potential of Icariin and Its Intestinal Metabolites via Inhibition of Intestinal UDP-Glucuronosyltransferases

**DOI:** 10.1155/2012/395912

**Published:** 2012-10-16

**Authors:** Yun-Feng Cao, Rong-Rong He, Jun Cao, Jian-Xing Chen, Ting Huang, Yong Liu

**Affiliations:** ^1^Key Laboratory of Contraceptives and Devices Research (NPFPC), Shanghai Engineer and Technology Research Center of Reproductive Health Drug and Devices, Shanghai Institute of Planned Parenthood Research, Shanghai 200032, China; ^2^Laboratory of Pharmaceutical Resource Discovery, Dalian Institute of Chemical Physics, Chinese Academy of Sciences, 457 Zhongshan Road, Dalian 116023, China; ^3^Pharmacy College, Jinan University, Guangzhou 510632, China; ^4^Occupational and Environmental Health Department, Dalian Medical University, Dalian 116044, China

## Abstract

Icariin is known as an indicative constituent of the *Epimedium* genus, which has been commonly used in Chinese herbal medicine to enhance treat impotence and improve sexual function, as well as for several other indications for over 2000 years. In this study, we aimed to investigate the effects of icariin and its intestinal metabolites on the activities of human UDP-glucuronosyltransferase (UGT) activities. Using a panel of recombinant human UGT isoforms, we found that icariin exhibited potent inhibition against UGT1A3. It is interesting that the intestinal metabolites of icariin exhibited a different inhibition profile compared with icariin. Different from icariin, icariside II was a potent inhibitor of UGT1A4, UGT1A7, UGT1A9, and UGT2B7, and icaritin was a potent inhibitor of UGT1A7 and UGT1A9. The potential for drug interactions *in vivo* was also quantitatively predicted and compared. The quantitative prediction of risks indicated that *in vivo* inhibition against intestinal UGT1A3, UGT1A4, and UGT1A7 would likely occur after oral administration of icariin products.

## 1. Introduction

**Figure 1:**
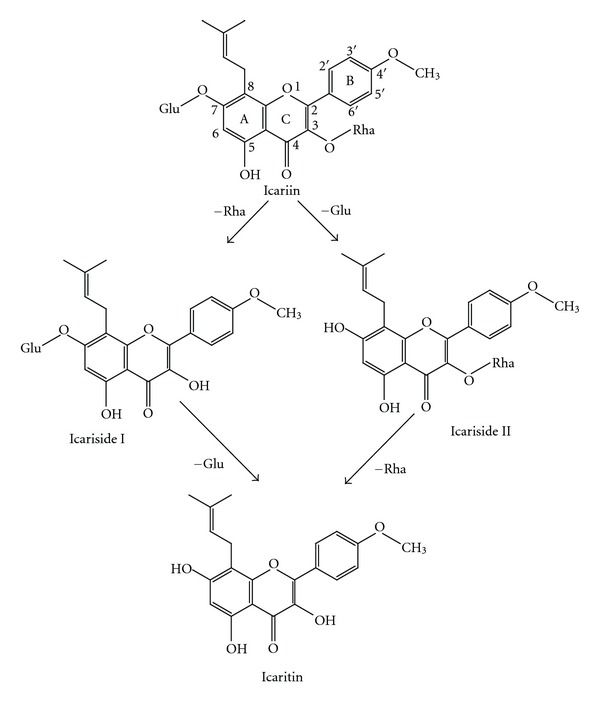
Structure of icariin and its intestinal metabolites. Rha, rhamnose; Glu, glucose.

Icariin (Figure 1), a typical flavonol glycoside, is known as an indicative constituent of the *Epimedium genus*, which is commonly known as horny goat weed or yin yang huo. Extracts from these plants are reputed to produce aphrodisiac effects and have been commonly used in Chinese herbal medicine to enhance treat impotence and improve sexual function, as well as for several other indications for over 2000 years [1]. It is thought that icariin is the primary active component of *Epimedium* extracts, as it has been shown to give various pharmacological effects, including immunoregulation [2], enhancement of cGMP levels in cavernous smooth muscle cells [3], enhancing the production of bioactive nitric oxide [4], as well as mimicking the effects of testosterone [5].

Herb-drug interactions have received increasing attention over the past few decades. To date, in many countries, numerous persons have ever taken icariin or *Epimedium* extracts however, little is known about the interactions between icariin and prescription drugs. Metabolizing enzyme-based drug-drug interactions (DDIs) constitute the major proportion of clinically important DDI [6]. Cytochrome P450 (CYPs) and UDP-glucuronosyltransferase (UGTs) isoforms are responsible for the metabolic clearance of more than 90% drugs clinically used [6]. The previous studies showed that icariin had no inhibitory effects on CYPs activities [7]. However, the effects of icariin on UGT activities have not been characterized. UGTs catalyze the conjugation of various endogenous substances and exogenous compounds. At least 22 human UGTs have been identified to date based on sequence homologies [8]. In humans, approximately 40–70% of all clinical drugs are subjected to glucuronidation reactions metabolized by UGTs [8, 9]. UGT-mediated DDI can potentially occur for many drugs, even resulting in enhanced adverse drug effects [10–12]. In fact, several significant DDIS have been clinically observed [13]. Thus, understanding the effects of icariin on UGT activities is important to ensure the safe administration of icariin.

As herbs are orally administered in most cases, the gastrointestinal tract serves principally as absorption site for absorption and first biotransformation site. Degradation of herb in the gastrointestinal tract is often observed [14]. Increasing attention has been paid to the role of herb metabolites in herb-drug interactions [15–19]. Previous study showed that icariin could be metabolized to three main metabolites icariside I, icariside II, and icaritin by the bacteria in rat intestine (Figure 1). Therefore, it is important to evaluate whether icariin and its intestinal metabolites possess the potential for exerting an influence on metabolic enzymes.

The aim of this study was to investigate the effects of icariin and its intestinal metabolites on the activities of human UGTs. Using a panel of recombinant human UGT isoforms, we found potent inhibition of icariin and its intestinal metabolites against several UGT isoforms. The potential for DDI *in vivo* was also quantitatively predicted and compared.

## 2. Materials and Methods

### 2.1. Chemicals

Icariin, icariside I, icariside II, and icaritin were purchased from Sichuan Victory Biotechnology Co., Ltd. (Chengdu, Sichuan, China). Trifluoperazine (TFP), 4-methylumbelliferone (4-MU), 4-methylumbelliferone-*β*-D-glucuronide (4-MUG), alamethicin (from Trichoderma viride), Tris-HCl, p-nitrophenol, androsterone, diclofenac, phenylbutazone, ascorbic acid, hecogenin, 7-hydroxycoumarin, and uridine 5′-diphosphoglucuronic acid (UDPGA) (trisodium salt) were purchased from Sigma-Aldrich (St. Louis, MO, USA). *β*-glucuronidase (from *Escherichia coli*, 103,000 unit/mL) were purchased from Wako Pure Chemical Industries (Osaka, Japan). All other reagents were of HPLC grade or of the highest grade commercially available.

### 2.2. Recombinant Human UGTs

A panel of recombinant human UGT supersomes (UGT1A1, UGT1A3, UGT1A4, UGT1A6, UGT1A7, UGT1A8, UGT1A9, UGT1A10, UGT2B4, UGT2B7, UGT2B15, and UGT2B17) expressed in baculovirus-infected insect cells were purchased from BD Gentest Corp. (Woburn, MA, USA). 

### 2.3. 4-MU Glucuronidation Assay

4-MU, a nonselective substrate of UGTs, was used as probe substrate for all UGTs except UGT1A4. Incubations with each individual enzyme were conducted using conditions previously described [10]. There was a 5 min preincubation step at 37°C before the reaction was started by addition of UDPGA. The incubation mixtures were then centrifuged at 20,500 ×g for 15 min to obtain the supernatant. Aliquots (20 *μ*L) were then analyzed by HPLC. The HPLC system (SHIMADZU, Kyoto, Japan) consisted of an SCL-10A system controller, two LC-10AT pumps, a SIL-10A auto injector, and a SPD-10A_VP_ UV detector. Chromatographic separation was achieved using a Kromasil ODS column (4.6 × 150 mm I.D., 5 *μ*m particle size) at a flow rate of 1 mL/min and UV detection at 316 nm. The mobile phase consisted of 10 mM KH_2_PO_4_, pH 2.7 (A) and acetonitrile (B). The following gradient was applied at a flow rate of 1 mL/min: 0–4 min 80% A and 20% B, 4.1–8 min 50% A and 50% B, 8.1–12 min, 30% A and 70% B. All experiments were performed in two independent experiments in duplicate. 

### 2.4. TFP Glucuronidation Assay

TFP was used as the substrate for UGT1A4. Trifluoperazine glucuronide (TFPG) formation was measured using a modification of the method reported [20]. The incubation mixture (200 *μ*L total volume) contained Tris–HCl buffer (50 mM, pH 7.4), UDPGA (5 mM), MgCl_2_ (5 mM), 50 *μ*g/mg protein alamethicin, 0.1 mg/mL for recombinant UGT1A4, and TFP. Reactions were initiated by the addition of UDPGA and incubations were performed at 37°C in a shaking water bath for 20 min. Incubations were terminated by the addition of 4% acetic acid/96% methanol (0.2 mL) and then centrifuged at 20,500 ×g for 15 min. A 40 *μ*L aliquot of the supernatant fraction was injected into the HPLC column.

Measurement of TFPG formation HPLC was performed using a SHIMADZU SCL-10A HPLC system (SHIMADZU, Kyoto, Japan) fitted with a Kromasil ODS column (4.6 × 150 mm I.D., 5 *μ*m particle size). A gradient mobile phase consisting initially of 30 : 70, mobile phase A (acetonitrile) verse mobile phase B (0.5% formic acid/water) was brought to a composition of 90 : 10 in 10 min which was held for 7 min, all at a flow rate of 1 mL/min. Column eluant was monitored by UV absorbance 256 nm. 

### 2.5. Inhibition of UGT Activity Assay

A typical incubation mixture contained recombinant human UGTs, 5 mM MgCl_2_, 5 mM UDPGA, 50 *μ*g/mg protein alamethicin, 50 mM Tris-HCl buffer (pH 7.4), and various probe substrates of UGTs in the absence or presence of different concentrations of inhibitors. Icariin, icariside I, icariside II, icaritin, and inhibitors were dissolved in DMSO. The final concentration of DMSO in the incubation system was 1% (v/v). Since 75–100 *μ*M are almost the highest plasma concentrations observed in patients of clinical drugs [21], the inhibition experiments with icariin were conducted at 1, 10, or 100 *μ*M. Incubations with 4-MU or TFP were performed at the concentration corresponding to the apparent *K*_*m*_ or *S*_50_ value reported for each isoform (110, 1200, 110, 15, 750, 30, 80, 1200, 350, 250, and 2000 *μ*M 4-MU for UGT1A1, UGT1A3, UGT1A6, UGT1A7, UGT1A8, UGT1A9, UGT1A10, UGT2B4, UGT2B7, UGT2B15, and UGT2B17, resp., or 50 *μ*M TFG for UGT1A4, resp.). Known UGT inhibitors were used as positive controls: diclofenac for UGT1A1, UGT1A6, UGT1A7, and UGT1A9; androsterone for UGT1A3, UGT2B7, and UGT2B15, phenylbutazone for UGT1A8 and UGT1A10, and hecogenin for UGT1A4, respectively [10]. There is no positive control reported available for UGT2B4 and UGT2B17. The negative controls are the incubation without UDPGA. Since their water solubility is poor, these tested chemicals and inhibitors were previously dissolved in DMSO for effective solubilization. The final concentration of DMSO in the incubation system was 1% (v/v). DMSO did not noticeably change the catalytic activity of UGTs at 1% (data not shown). There was a 5 min preincubation step at 37°C before the reaction was started by the addition of UDPGA. Incubation times were 120 min for UGT1A1, UGT1A10, UGT2B4, UGT2B15, and UGT2B17, 75 min for UGT1A3, and 30 min for UGT1A4, UGT1A6, UGT1A7, UGT1A8, and UGT1A9. The reactions were quenched by adding 100 *μ*L acetonitrile and internal standard. The incubation mixtures were then centrifuged at 20,500 ×g for 15 min to obtain the supernatant. An aliquot of the supernatant was used for HPLC analysis as described above. All experiments were performed in two independent experiments in duplicate.

### 2.6. Inhibition Kinetics Analysis

Inhibition constant (*K*_*i*_) values were determined using various concentrations of 4 MU or TFP in the presence or absence of icariin. Inhibition data from experiments were graphically represented by Dixon plots. *K*_*i*_ values were calculated by nonlinear regression using the equations for competitive inhibition (1), noncompetitive inhibition (2), or mixed inhibition (3), [22]

(1) $$\begin{array}{c} v\;=\frac{V_{\max}S}{K_{m}\left(1+\left(I/K_{i}\right)\right)+S}, \end{array}$$

(2) $$\begin{array}{c} v\;=\frac{V_{\max}S}{\left(K_{m}+S\right)\left(1+\left(I/K_{i}\right)\right)}, \end{array}$$

(3) $$\begin{array}{c} v\;=\frac{V_{\max}S}{K_{m}\;\left(1+\left(I/K_{i}\right)\right)+S\left(1+\left(I/\alpha K_{i}\right)\right)}, \end{array}$$

where *v* is the velocity of the reaction; *S* and *I* are the substrate and inhibitor concentrations, respectively; *K*_*i*_ is the inhibition constant describing the affinity of the inhibitor for the enzyme; *K*_*m*_ is the substrate concentration at half of the maximum velocity (*V*_max⁡_) of the reaction; *α* reflects the effect of inhibitor on the affinity of the enzyme for its substrate. The type of inhibition was determined from the enzyme inhibition models. Goodness of fit to kinetic and inhibition models was assessed from the *F* statistic, *r* ^2^ values, parameter standard error estimates, and 95% confidence intervals. Kinetic constants are reported as the mean value ± standard error of the parameter estimate. IC_50_ values (concentration of inhibitor that reduces enzyme activity by 50%) were determined by GraphPad Prism5 software (GraphPad Software, Inc., La Jolla, CA, USA).

### 2.7. Calculation of the Concentrations of Icariin in Gut Lumen and Blood

Since the concentrations of icariin in human gut lumen are not available, the values were calculated based on physiological parameters of human and the dosage of a single oral administration of a Chinese traditional decoction of *Epimedium pubescens* in human volunteers. 

Based on the assumption that the possible maximum concentrations of icariin in gut lumen were the ratio of oral administered dose excluding of the fraction absorbed into blood to the volume of gut lumen, the possible maximum concentrations of icariin in human gut lumen after a single oral administration of *Epimedium pubescens* decoction were estimated according to (4),

(4) $$\begin{array}{c} C_{L}=\text{Dose}\frac{\left(1-F_{a}\right)}{\text{MW}\cdot V_{L}}, \end{array}$$

where *C*_*L*_ is the concentration of icariin in gut lumen after a single oral administration of a Chinese traditional decoction of *Epimedium pubescens* in human volunteers, *F*_*a*_ is the extent of absolute oral bioavailability of icariin, MW is the molecular weight of icariin, and *V*_*L*_ is the average human gut volume. The reported *V*_*L*_ was 1.65 L/70 kg [23]. The reported oral bioavailability of icariin was 0.12 [24].

The concentrations of icariin intestinal metabolites in human gut lumen are not available, but it has been reported that about 70% icariin will be transformed into its intestinal metabolites in the intestinal lumen [25]. Then the concentrations of icariside I, icariside II, and icaritin in human gut lumen were calculated based on the above calculated concentration of icariin and the ratio of their concentrations in blood after oral administration of icariin [26]. Here we arbitrarily assumed the ratio in rat equaled to that in human blood, and the values of their oral bioavailability were consistent.

The possible maximum concentrations of icariin, icariside I, and icariside II in human blood were calculated with the reported icaritin concentration (1.5 nM) after a single oral administration of *Epimedium pubescens* decoction [27] and the ratio of their concentrations in blood after oral administration of icariin [26].

### 2.8. Prediction of Risks of *In Vivo* Inhibition of Icariin on UGTs

Risks of *in vivo* inhibition of icariin were estimated by calculating the ratios of [*I*]/*K*_*i*_, where [*I*] represented the possible *in vivo* concentration of icariin. For reversible inhibition, if the ratio of [*I*]/*K*_*i*_ was greater than 1, *in vivo* inhibition on the UGTs would likely occur [28].

## 3. Results

### 3.1. Inhibition of UGTs Activities by Icariin in Recombinant Human UGTs

As shown as Table 1, icariin exhibited moderate inhibitory effect against UGT1A3 activity with an IC_50_ value of 12.4 ± 0.1 *μ*M and also weak inhibition against UGT1A4 activity. It is interesting that the intestinal metabolites of icariin exhibited a different inhibition profile compared with icariin. Icariside II inhibited UGT1A4, UGT1A7, UGT1A9, and UGT2B7 activities, with an IC_50_ value of 2.9 ± 0.1 *μ*M, 2.8 ± 0.1 *μ*M, 2.4 ± 0.1 *μ*M, and 12.5 ± 0.1 *μ*M, respectively. Icaritin exerted potent inhibition against UGT1A7 and UGT1A9, with an IC_50_ value of 0.3 ± 0.0 *μ*M, and 1.5 ± 0.1 *μ*M, respectively.

### 3.2. Inhibition Kinetic Analysis in Recombinant UGTs

**Figure 2:**
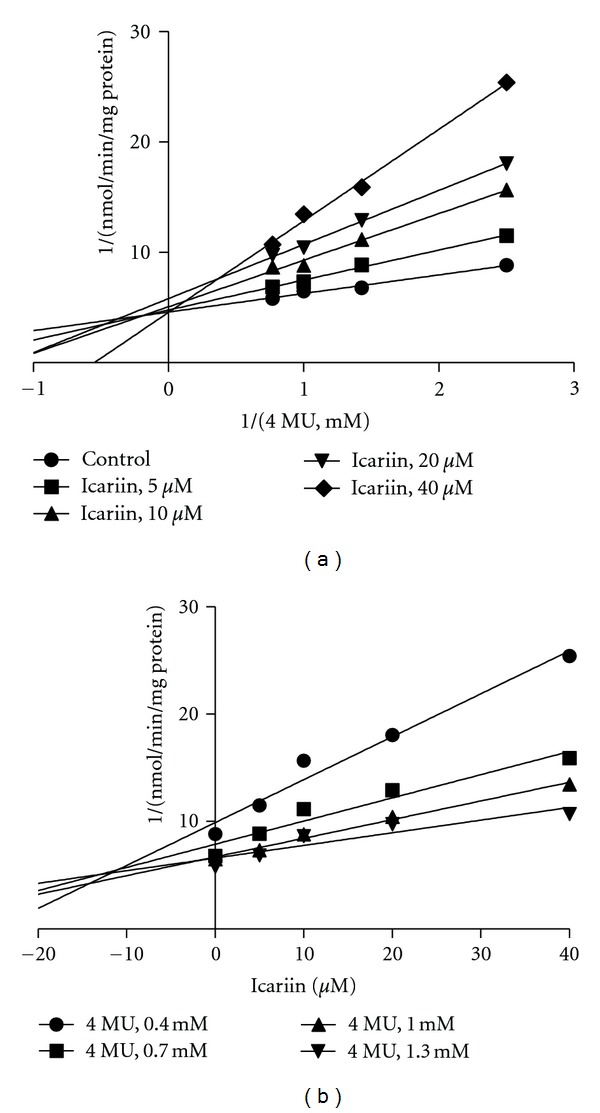
Representative Lineweaver-Burk plots (a) and Dixon plots (b) of the effects of icariin on 4-MU glucuronide formation in recombinant UGT1A3. Reactions were performed as described in Materials and Methods. All data points shown represent the mean of duplicate measurements.

**Figure 3:**
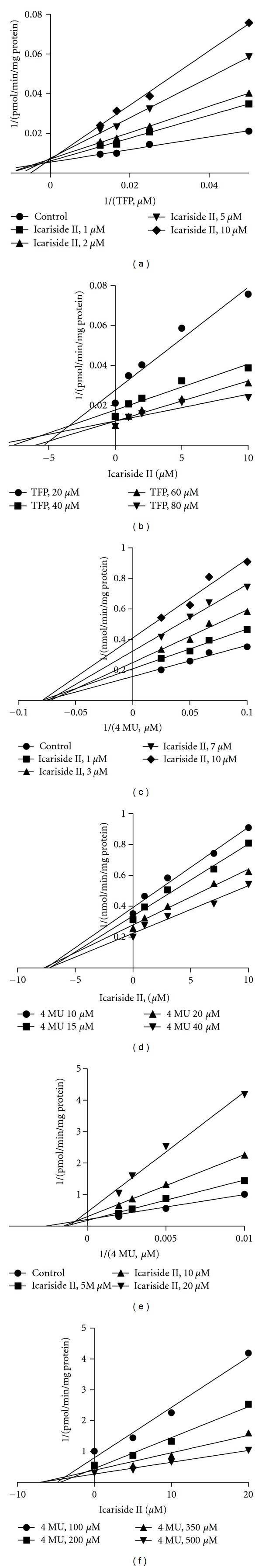
Representative Lineweaver-Burk plots (a, c, e) and Dixon plots (b, d, f) of the effects of icariside II on UGT1A4 (a and b), UGT1A7 (c and d), and UGT2B7 (e and f) in recombinant UGTs. Reactions were performed as described in Materials and Methods. All data points shown represent the mean of duplicate measurements.

**Figure 4:**
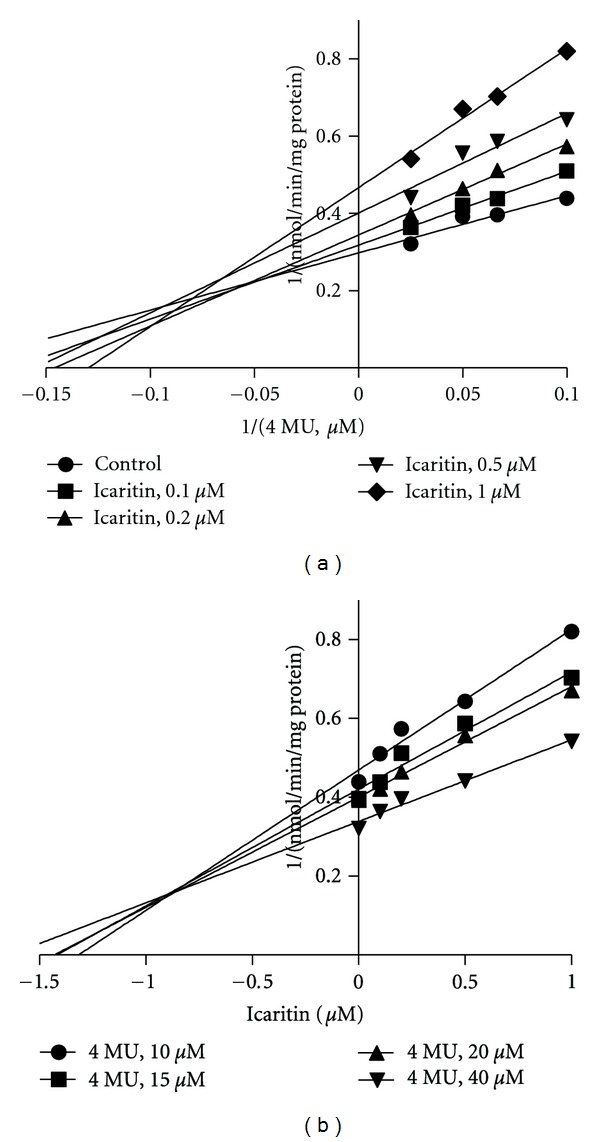
Representative Lineweaver-Burk plots (a) and Dixon plots (b) of the effects of icaritin on 4-MU glucuronide formation in recombinant UGT1A7. Reactions were performed as described in Materials and Methods. All data points shown represent the mean of duplicate measurements.

Kinetic experiments were performed to further characterize the inhibition of UGT activities by icariin, icariside II, and icaritin. Icariin strongly inhibited the formation of 4-MUG by UGT1A3. The representative Lineweaver-Burk plots for the inhibition of 4-MUG formation by icariin (Figure 2(a)) and analysis of the parameters of the enzyme inhibition model suggested that the inhibition types were competitive. Based on nonlinear regression analysis and Dixon plots presented in Figure 2(b), icariin showed competitive inhibition against the formation of 4-MUG with *K*_*i*_ of 8.0 ± 1.4 *μ*M in recombinant UGT1A3. Icariside II exhibited potent competitive inhibition against UGT1A4 with *K*_*i*_ of 1.9 ± 0.3 *μ*M (Figures 3(a) and 3(b)). It also exerted noncompetitive inhibition against UGT1A7 with *K*_*i*_ of 6.2 ± 0.5 *μ*M (Figures 3(c) and 3(d)) and mixed inhibition against UGT2B7 with *K*_*i*_ of 8.2 ± 1.5 *μ*M and *α* of 3.3 (Figures 3(e) and 3(f)). Icaritin exerted mixed inhibition against UGT1A7 with *K*_*i*_ of 0.7 ± 0.2 *μ*M and *α* of 2.7 (Figures 4(a) and 4(b)). 

### 3.3. The Calculated Concentrations of Icariin and Its Intestinal Metabolites in Blood and Gut Lumen

The possible maximum concentrations of icariin, icariside I, icariside II, and icaritin in human gut lumen after a single oral administration of *Epimedium pubescens* decoction were calculated to be about 9.9 *μ*M, 0.2 *μ*M, 3.7 *μ*M, and 3.8 *μ*M, respectively. The possible maximum concentrations of icariin, icariside I, icariside II, and icaritin in human blood after a single oral administration of *Epimedium pubescens* decoction were calculated to be about 1.3 nM, 0.1 nM, 1.5 nM, and 1.5 nM, respectively.

### 3.4. Quantitative Prediction of Risks of *In Vivo* Inhibition on UGTs by Icariin

Risks of *in vivo* inhibition on UGT1A3 by icariin, icariside II, and icaritin were estimated by calculating the ratios of [*I*]/*K*_*i*_. After a single oral administration of *Epimedium pubescens* decoction, the ratio of [*I*]/*K*_*i*_ was 1.2 for the inhibition of icariin against intestinal UGT1A3. For icariside II, the values were 1.9 for intestinal UGT1A4, respectively. For icaritin, the ratio was 5.4 for intestinal UGT1A7. For reversible inhibition, if the ratio of [*I*]/*K*_*i*_ were greater than 1, *in vivo* inhibition on the UGTs would likely occur [28]. Thus, *in vivo* inhibition against intestinal UGT1A3, UGT1A4, and UGT1A7 would likely occur after a single oral administration of *Epimedium pubescens* decoction.

As for hepatic UGTs, the values were negligible.

## 4. Discussion

DDIs caused by inhibition of drug-metabolizing enzymes receive considerable attention due to their clinical relevance. As a result of increased understanding, the use of *in vitro* approaches to predict aspects of human drug metabolism and pharmacokinetics *in vivo* has found increasing acceptance in recent years.

Our data offer *in vitro* evidence that icariin and its intestinal metabolites are potent inhibitors of several UGT isoforms. We found that icariin exhibited potent inhibition against UGT1A3. It is interesting that the intestinal metabolites of icariin exhibited a different inhibition profile compared with icariin. Different from icariin, icariside II was a potent inhibitor of UGT1A4, UGT1A7, UGT1A9, and UGT2B7, and icaritin was a potent inhibitor of UGT1A7 and UGT1A9. UGT1A3 is responsible for the metabolism of several endogenous and exogenous substrates, including bile acid, naringenin, quercetin, estrone, anthraquinones, naproxen, opioids, ketoprofen, ezetimibe, 7-hydroxycoumarins, losartan, candesartan, and zolarsartan [29, 30]. UGT1A4 can catalyze the tertiary amines including imipramine, amitriptyline, doxepin, promethazine, chlorpromazine, loxapine, and cyproheptadine [29]. UGT1A7 is involved in the glucuronidation of dulcin, SN-38, acetaminophen, mycophenolic acid, and so on [13]. UGT1A9 is involved in the glucuronidation of a number of drugs, including flavopiridol, mycophenolic acid, propofol, acetaminophen, and others [13]. UGT2B7 is the most commonly listed enzyme (35%) involved in glucuronidation of the top 200 prescribed drugs in the United States in 2002 [9]. Therefore, the potent inhibition of UGTs activities by icariin and its intestinal metabolites can modulate the metabolism of numerous drugs cleared by UGTs.

The expression levels of UGT1A3, UGT1A4, UGT1A9, and UGT2B7 are high in human liver, whereas UGT1A3, UGT1A4, UGT1A7, and UGT2B7 are highly expressed in the gastrointestinal tract [13, 31, 32]. In view of the low levels of icariin and its intestinal metabolites in blood, icariin is unlikely to cause a clinically significant DDI through inhibition of hepatic glucuronidation after oral administration. However, the quantitative prediction of risks of *in vivo* inhibition on intestinal UGTs by icariin and its intestinal metabolites indicated that *in vivo* inhibition against intestinal UGT1A3, UGT1A4, and UGT1A7 would likely occur after a single oral administration of *Epimedium pubescens* decoction. UGTs in the gastrointestinal tract may contribute significantly to the first-pass metabolism of orally administered drugs that undergo glucuronidation [13]. These results showed that icariin might exert an influence on the glucuronidation and first-pass metabolism of some drugs orally administered. In addition, *in vitro* data tend to underestimate inhibition of drug glucuronidation *in vivo* [20], and the pharmacokinetic parameters used here to calculate concentrations are mean values of the parameters reported, but interindividual variability is large. So the actual effects of icariin might be more potent than those calculated here. 

This finding also offers new experimental evidence for the opinion that the biotransformation of herb in the gastrointestinal tract could play a key role in the herb-associated DDI [17, 18]. Our data shows that the degradation products of herb by gastrointestinal factors may exhibit distinct effects on metabolic enzymes compared to naturally occurring components. Additional attention should be paid on the effects of intestinal metabolites of herbs on the metabolic enzymes during the safety evaluation of herbal products. Our results might be helpful to clinically safe administration of icariin, but further DDI studies with associated drugs will need to be performed to evaluate whether this *in vitro* phenomenon also occurs *in vivo*. 

In conclusion, Icariin and its intestinal metabolites were found to be potent inhibitors of several UGT isoforms. The *in vivo* inhibition against intestinal UGT1A3, UGT1A4, and UGT1A7 would likely occur after a single oral administration of *Epimedium pubescens* decoction. The present findings shed light on the mechanisms underlying clinically significant DDI associated with icariin and also provide the basis for further *in vivo* studies investigating the DDI potential between icariin with UGT substrates.

## Figures and Tables

**Table 1:** The IC_50_ values for the inhibition of icariin and its intestinal metabolites on UGT activities^a^.

IC_50_(*μ*M)	Icariin	Icariside I	Icariside II	Icaritin
UGT1A1	>100	>100	>100	>100
UGT1A3	12.4 ± 0.1	>100	>100	>100
UGT1A4	42.8 ± 2.4	>100	2.9 ± 0.1	>100
UGT1A6	>100	>100	>100	>100
UGT1A7	>100	>100	2.8 ± 0.10	0.3 ± 0.0
UGT1A8	>100	>100	>100	>100
UGT1A9	>100	>100	2.4 ± 0.1	1.5 ± 0.1
UGT1A10	>100	>100	>100	>100
UGT2B4	>100	>100	>100	>100
UGT2B7	>100	>100	12.5 ± 0.1	>100
UGT2B15	>100	>100	>100	>100
UGT2B17	>100	>100	>100	>100

^a^Data were showed as mean ± SD. All experiments were separately performed in duplicate for three times.
